# On-surface synthesis

**DOI:** 10.1038/s42004-024-01359-0

**Published:** 2024-12-09

**Authors:** Jennifer MacLeod, Alexander Saywell, Ping Yu

**Affiliations:** 1 School of Chemistry and Physics and Centre for Materials Science, Queensland University of Technology (QUT), Brisbane, Australia; 2 School of Physics & Astronomy, University of Nottingham, Nottingham, UK; 3 School of Physical Science and Technology, ShanghaiTech University, Shanghai, China

**Keywords:** Surface chemistry, Synthesis and processing, Synthesis of graphene

## Abstract

*Communications Chemistry* is pleased to introduce a Collection of research works focused on recent developments within the interdisciplinary field of on-surface synthesis. Here, the Guest Editors highlight key themes and look towards the future of this research field.

On-surface synthesis is an emerging research field focused on creating atomically precise low-dimensional nanostructures with unique properties through controlled organic chemistry reactions on surfaces. The well-defined environment provided by ultra-high vacuum (UHV) conditions is often utilised and powerful surface science characterization techniques are leveraged to probe the interactions and reactivity of surface-confined molecules. In recent decades, significant progress has been made in understanding on-surface reactions using ab initio theories and applying this knowledge to synthesize and characterize nanographene, one-dimensional polymers (including graphene nanoribbons), and two-dimensional covalent/metal-organic frameworks. To support the ongoing work in this area, we are pleased to present a variety of articles that showcase advancements in this exciting field. These papers cover three main themes: 1. Probing the mechanisms of on-surface synthesis; 2. On-surface synthesis as a route to preparing graphitic materials; and 3. From novel materials to devices and applications.

## Probing the mechanisms of on-surface synthesis

The study of surface-confined reactions provides an ideal framework to characterise the mechanistic processes which underpin a range of synthetic pathways. While many on-surface reactions have analogues within solution-phase synthesis, the conditions employed ‘on-surface’ enable reaction steps which cannot yet be achieved in solution. Techniques such as scanning probe microscopy (SPM) and photoelectron spectroscopies (PES) provide chemical and structural information on initial, intermediate and product states within a reaction, by combining the spatial, sub-molecular and bond-resolved resolution provided by SPM with the chemical sensitivity of PES. Importantly, these kinds of studies are likely to provide the insight required to enhance selectivity and efficiency for a range of reactions. Continued development of on-surface synthesis approaches will facilitate the design of molecular-based materials tailored to exhibit bespoke catalytic, (opto-)electronic, and magnetic properties.

This Collection showcases recent work detailing reaction mechanisms key to the formation of surface-confined metal-organic polymers (10.1038/s42004-020-00402-0)^1^ and covalent polymers (10.1038/s42004-023-01073-3)^2^, as well as highlighting novel methodologies to influence, and potentially control, on-surface synthesis, such as utilising electric field mediated polymerization at the solution-solid interface (10.1038/s42004-024-01187-2)^3^, and employing enantioselective approaches (10.1038/s42004-024-01137-y and 10.1038/s42004-021-00488-0)^4,5^. In parallel with such studies, developments in instrumentation and analysis protocols provide access to alternate measurement environments, e.g. sub-angstrom structural characterisation of molecule–surface systems performed at room temperature (10.1038/s42004-023-01093-z)^6^, and can provide insight into key structural properties. Indeed, the combination of atomic force microscopy with infrared spectroscopy provides information of molecular conformation (10.1038/s42004-023-01036-8)^7^, including details of electronic properties, e.g. the *p*-orbital Kagome bands observed in a two-dimensional metal-organic framework (10.1038/s42004-023-00869-7)^8^.

## On-surface synthesis as a route to preparing graphitic materials

Graphene-based nanostructures, such as graphene nanoribbons (GNRs) and nanoflakes, have attracted significant interest in chemistry and materials science due to their intriguing properties. On-surface synthesis provides a promising approach for fabricating functionalized graphene-based nanostructures with atomic precision through specifically designed precursors. The research featured in this section of the Collection focuses on the synthesis of functionalized graphene-based nanostructures. It covers nitrogen-doped porous nanographene, achieved through preparation strategies such as synthesizing nitrogen-doped porous carbon nanoribbons via stepwise on-surface polymerization (10.1038/s42004-024-01123-4)^9^ as well as generating nitrogen-doped nanographene with an [18] annulene pore via sequential debromination(10.1038/s42004-023-01023-z)^10^. The fabrication of nanoporous nanoribbons upon generating arrays of [18]-annulene pores at the nanostructure edges by phenyl migration is also highlighted (10.1038/s42004-024-01284-2)^11^. Additionally, this section shows chevron-type GNRs obtained by growing high-quality GNRs with fluorine-bearing chevron-type GNR precursors (10.1038/s42004-024-01253-9)^12^, as well as edge-functionalized graphene-based materials realized by extending the edges of 7-AGNRs with pyridine rings (10.1038/s42004-024-01344-7)^13^. Moreover, a Review article of progress in the on-surface synthesis of nanoporous graphene is also highlighted (10.1038/s42004-024-01222-2)^14^.

## From novel materials to devices and applications

The dimensionally constrained, controlled environment of on-surface synthesis provides an ideal playground for the formation of new materials tailor-made for applications, as well as the elucidation of the mechanisms underlying industrially relevant processes, such as the behaviour of molecular fragments trapped at catalytic surfaces (10.1038/s42004-020-00444-4)^15^, or how interaction with carefully-selected molecules may increase the activity of surface adatoms for specific processes (10.1038/s42004-023-01066-2)^16^.

In terms of new materials, an exciting advantage of on-surface synthesis is to allow access to organic molecules that may be difficult to synthesise through other approaches, for example, due to limitations on solubility or challenges in purification. The examples of macrocyclic molecular synthesis presented in this Collection provide insight into the (sometimes surprising) benefits of working on-surface: a mixed-product synthesis of conjugated tetraphenylethylene macrocycles exhibits self-selection into mono-component 2D self-assemblies, in a sort of auto-purification process (10.1038/s42004-022-00794-1)^17^, and on-surface dinitrile tetramerization can lead to products with bespoke conjugation and related properties (10.1038/s42004-024-01351-8)^18^.

However, an ongoing non-trivial challenge for on-surface synthesis lies in the integration of products into application-ready architectures. For example, materials crafted for specific electronic or optical properties will generally need to be removed from a metallic substrate to avoid hybridisation and to ensure that the substrate does not contribute to systems properties, such as by short-circuiting the synthesised material. A number of approaches are available to avoid or mitigate this challenge, but direct synthesis of monolayer materials at the liquid/gas interface, e.g., by using a Langmuir–Blodgett approach, is an elegant solution that allows for facile Langmuir–Schafer transfer of the material into a device geometry (10.1038/s42004-023-01081-3)^19^. While this approach sacrifices some of the advantages of, e.g., in-vacuum synthesis, it clearly provides key benefits in terms of the usability of products.

## Outlook

The work presented within this Collection highlights some of the recent advances made within the field of on-surface synthesis. A noteworthy feature of the Collection is the plurality of applications for on-surface synthesis approaches. Studying surface-confined molecules has expanded the boundaries of knowledge on a fundamental level, specifically with respect to molecular interactions and on-surface chemistry, and has facilitated the design and characterisation of novel materials (including a range of innovative low-dimensional graphene-based nanostructures). Moving forward, the field is providing motivation to develop methodologies for incorporating on-surface synthesised materials into devices for a range of applications. Finally, we hope that this Collection will inspire new avenues of interdisciplinary research into the fundamental science, novel materials, and applications made accessible by on-surface synthesis.
